# Adult Hippocampal Neurogenesis Modulation by the Membrane-Associated Progesterone Receptor Family Member Neudesin

**DOI:** 10.3389/fncel.2018.00463

**Published:** 2018-11-26

**Authors:** Ashley Novais, Alberto Silva, Ana Catarina Ferreira, Ana Mendanha Falcão, Nuno Sousa, Joana Almeida Palha, Fernanda Marques, João Carlos Sousa

**Affiliations:** ^1^Life and Health Sciences Research Institute (ICVS), Neuroscience Domain, School of Medicine, University of Minho, Braga, Portugal; ^2^ICVS/3B’s-PT Government Associate Laboratory, Guimarães, Portugal

**Keywords:** newborn neurons, NENF, dentate gyrus, delta GABA A receptor, progesterone

## Abstract

Neudesin (Neuron-derived neurotrophic factor, NENF), a membrane-associated progesterone receptor family (MAPR) member, is a neuron secreted protein with neurotrophic properties during embryonic stages. However, its role in the adult brain is still poorly addressed. In this study we have used neudesin-null (Nenf^−/−^) mice and performed a characterization of the proliferation state of the adult neurogenic niches, the adult subventricular zone (SVZ) and the hippocampus subgranular zone (SGZ). Nenf^−/−^ males did not presented any deficits in proliferation in the SVZ neither *in vivo* nor *in vitro*. On the other hand a decrease in cell proliferation in the SGZ was observed, as well as a decrease in the number of newborn neurons in the dentate gyrus (DG) that was accompanied by impaired context discrimination in a contextual fear conditioning (CFC) task. Since NENF neurotrophic action is suggested to occur via the formation of a progesterone stability complex for the activation of non-genomic cascade, we further evaluated progesterone metabolism in the absence of NENF. Interestingly, expression of progesterone catabolic rate-determining enzyme, 5-α-reductase was upregulated in the DG of Nenf^−/−^, together with a significant increase in the expression of the δGABA_A_ receptor gene, involved in DG tonic inhibition. Taken together, these findings add *in vivo* evidence on the neurotrophic properties of NENF in the adult brain. Furthermore, the mechanism of action of NENF in this process might implicate neurosteroids modulation, at least in the DG.

## Introduction

Neuron-derived neurotrophic factor (NENF), commonly known as neudesin, is a secreted neuronal protein with 21 KDa and 171 aminoacids (Kimura et al., 2005). Since neudesin presents a cytochrome b5-like heme/steroid binding domain in its primary structure, it is classified as a member of the membrane-associated progesterone receptor family (MAPR; Kimura et al., 2008). While NENF is highly expressed particularly in neurons in embryonic stages in the brain and spinal cord, in the adult mice NENF is also expressed in other organs besides the nervous system, namely in the heart, lung and kidneys (Kimura et al., 2005). In the embryonic brain cortical formation, NENF expression was found in the pre-plate region (where post-mitotic neurons are located), but was not found in regions of precursors proliferation and migration (Kimura et al., 2006). *In vivo*, in the central nervous system (CNS) neurons differentiate between E13.5 and E16.5 while glia appears later in development; NENF is expressed in all of these embryonic stages (Kimura et al., 2006). The specific pattern of NENF expression and secretion by neurons suggests that it plays a role exclusively in neurons but not in other neural cell types. In fact, *in vitro*, neudesin was shown to promote proliferation and differentiation of neural precursors into a neuronal lineage as well as to promote the survival of mature neurons (Kimura et al., 2005, 2006). Given these properties elicited by recombinant NENF *in vitro* and the pathways activated, this protein was proposed to be a neurotrophic factor (Kimura et al., 2005).

While *in vitro* evidence exists for a potential neurotrophic action of NENF in the proliferation and differentiation of embryonic neural precursor cells, NENF action in the adult brain is poorly understood. Until now, NENF’s direct action in the adult brain seems to be exclusive of the hypothalamus, where NENF expression is high (Petersen et al., 2013). In fact, NENF was identified as a novel player in the regulation of food intake in a large-scale screening, as one of the four genes with the highest expression in the hypothalamus of a diet-induced obese model (Byerly et al., 2013), and was later associated with obesity-metabolic dysfunction via modulation of sympathetic activity (Ohta et al., 2015). Interestingly, we have previously shown that the constitutive ablation of NENF in mice results in an anxious-like behavior and morphologic atrophy of granule neurons in the hippocampal dentate gyrus (DG; Novais et al., 2013). Surprisingly, given the described neurotrophic action of NENF, until now no study explored the effects of NENF neurotrophic properties in the classic adult neurogenic niches of the brain, the subventricular zone (SVZ) and the subgranular zone (SGZ) of the hippocampus. For both SVZ and SGZ neurogenic niches, several molecular players have a role in the modulation of the neural stem cell population, and NENF might be one of these factors.

NENF mechanism of action is still not entirely understood. Inhibition of Gi/Go-coupled receptors *in vitro* blocks NENF neurotrophic activity, suggesting that NENF might exert its effects via non-genomic progesterone receptors (Kimura et al., 2008). Given that binding of steroid hormones to their membrane receptors is allowed by a complex of proteins that provide stability to the receptors, it was hypothesized that NENF might promote stability to progesterone in the activation of progesterone membrane receptors (Kimura et al., 2013). Supporting this hypothesis is the fact that NENF belongs to the MAPR family and shares 40% structural homology with the progesterone receptor membrane component 1 (PGRMC1). Noteworthy, progesterone alone was observed to be able to promote neural precursors proliferation through PGRMC1 (Liu et al., 2009). Nevertheless, one of the significant contributions of progesterone for hippocampal neurogenesis and function is its metabolization into neuro-active steroids such as allopregnanolone (Zhang et al., 2015; Karout et al., 2016). Progesterone metabolites (through 5α-reductase) play an important role in keeping excitability/inhibition balance and, particularly in the hippocampal DG, allopregnanolone allosteric binding to delta^+^ GABA A receptors (GABRD gene) modulates tonic inhibition, essential for the “dentate gate” perforant pathway, and essential for a correct hippocampal function (Maguire et al., 2005; Maguire and Mody, 2007).

In this study, using a mouse model with constitutive ablation of NENF gene, we assessed the role of NENF in the regulation of adult neurogenesis. Furthermore, we explored if NENF plays a role in progesterone metabolism in the DG.

## Materials and Methods

### Animals

A mouse strain with a targeted deletion of the *NENF* gene, provided by Merck-Serono under a material transfer agreement, was used, as previously reported (Novais et al., 2013). All experiments were performed in 2-month old male mice lacking the expression of NENF and the respective wild-type littermate controls in a C57BL/6J mouse background. Littermate controls (Nenf^+/+^) and neudesin-null (Nenf^−/−^) animals were obtained by crossing heterozygous animals. Animals, housed in groups of five, were maintained under 12 h light/dark cycles at 22 ± 1°C, 55% humidity and fed with regular rodent’s chow and tap water *ad libitum*. The Portuguese national authority for animal experimentation, Direção Geral de Veterinária, approved this study (permission ID: DGV9457). All experiments were performed following the guidelines for the care and handling of laboratory animals, as described in the Directive 2010/63/EU of the European Parliament and of the Council. A different set of animals was used for fear conditioning and immunohistochemistry experiments.

### Administration of 5-bromo-2′-Deoxyuridine (BrdU) for Proliferation Label Retaining Cells Assessment

5-bromo-2′-deoxyuridine (BrdU) is a thymidine analog that incorporates in the DNA during the S phase of the mitotic cycle. To label proliferative cells in the SVZ and SGZ, mice were intraperitoneally (i.p.) injected with 50 mg/kg of BrdU (Sigma Aldrich, St. Louis, MO, USA) 24 h prior to sacrifice. To label newly born granular neurons at the DG, animals were i.p. injected with BrdU twice a day for five consecutive days and sacrificed 7 weeks later (Deng et al., 2013; Ferreira et al., 2017). During this period of chase, neuroblasts proliferate and mature, thus BrdU positive cells co-label with a mature neuronal marker of integrated newborn granular neurons in the DG circuitry, represent not only the morphologically mature cells but also functionally mature DG granule cells (van Praag et al., 2002; Zhao et al., 2006; Kee et al., 2007).

### Immunohistochemistry and Immunofluorescence

For immunohistochemistry, 2-month-old littermate control and neudesin-null mice were used (*n* = 7 in each group). Animals were transcardially perfused, under deep anesthesia [ketamine hydrochloride (150 mg/Kg) with medetomidine (0.3 mg/Kg)], with 4% of paraformaldehyde (PFA) in 0.01 M phosphate-buffered saline (PBS). Brains were removed, postfixed for 1 h in 4% PFA solution, cryoprotected in 30% sucrose solution for 12 h, and then embedded in optimal cutting temperature compound (ThermoFisher Scientific, Waltham, MA, USA), snap-frozen in liquid nitrogen and kept at −20°C until sectioning in a cryostat. Immunohistochemistry was performed on coronal 20 μm thick serial sections, pre-treatment of the brain tissue involved antigen retrieval in pre-heated 95–100°C citrate buffer (10 nM, pH6; Sigma) bath for 15 min; additionally for BrdU immunostaining, the sections received a pre-treatment for DNA denaturation with HCl for 30 min at room temperature (RT). Slides were incubated with an anti-BrdU primary antibody at a dilution of 1:50 (mouse anti-bromodeoxyuridine, Clone Bu20a, DAKO, Barcelona, Spain) and an anti-Ki67 primary antibody at a dilution of 1:300 (rabbit; Novocastra, Newcastle, UK). These were detected using a secondary antibody with the Ultravision Detection System (Lab Vision, Freemont, CA, USA), and the reaction developed with 3,3′-diaminobenzidine substrate (Sigma). The sections were subsequently counterstained with hematoxylin.

For immunofluorescence analysis, six littermate control and six neudesin-null mice were used. Immunofluorescence analysis was performed on fixed 20 μm coronal sections, pre-treatment using antigen retrieval and DNA denaturation (for BrdU) was also performed. The sections were further incubated with the following primary antibodies: doublecortin (DCX; rabbit polyclonal, Abcam, Cambridge, UK) at a dilution of 1:300, BrdU (rat anti-BrdU, BU1/75 clone, Abcam) at a dilution of 1:100, Calbindin (Calb; rabbit polyclonal, Abcam) at a dilution of 1:300 and neudesin at a dilution of 1:50 (NENF; rabbit polyclonal; Sigma). Fluorescent secondary antibodies (Invitrogen, Carlsbad, CA, USA), anti-rabbit (for DCX, Calb and NENF) and anti-rat (for BrdU), were used to detect the primary antibodies at a dilution of 1:500. To label the nucleus, incubation with 4’,6-diamidino-2-phenylindole (DAPI; Sigma) at a dilution of 1:200 was performed. Antibodies were diluted in PBS-0.5% Tween with 10% fetal bovine serum (FBS) and incubated overnight at RT, for the primary antibodies, and 2 h at RT, for the secondary antibodies.

### Cellular Density Counting

The number of BrdU positive cells was assessed in the neurogenic niches using an Olympus BX51 microscope (Olympus, Hamburg, Germany) associated with the Visiopharm Integrator system (VIS) software (version 2.12.3.0). Coronal sections comprised coordinates between Bregma 0.86 mm and 0.14 mm for the SVZ (Falcão et al., 2013), −1.22 mm to −2.30 mm for the dorsal SGZ and −2.80 mm to −3.88 mm for the ventral SGZ. These boundaries were chosen as a representation of the septal and temporal poles of the hippocampus, known to be distinct in morphology and connectivity (Pinto et al., 2015). The number of BrdU positive cells and Ki67 positive cells were normalized for the respective area (mm^2^).

### Neurospheres Clonal Expansion Assay

Hippocampi DG and SVZ from 3 days to 6 days old Nenf^+/+^ and Nenf^−/−^ male mice were carefully dissected and meninges and choroid plexus removed. Tissue was triturated in DMEM F12 (Gibco, Rockville, MD, USA) with 10% FBS with the help of a P1000 micropipette; upon completion of tissue dissociation, the cell suspension was centrifuged at 900 rpm and the supernatant carefully discarded. Single cells obtained were plated in DMEM F12 media supplemented with GlutaMAX (Gibco), 100 U/ml penicillin, 100 μg/ml streptomycin (Gibco), 1% B27 (Gibco), 10 ng/ml epidermal growth factor (Gibco), and 10 ng/ml basic fibroblast growth factor (Gibco). Neurospheres were allowed to form and grow at 37°C in a 95% air-5% CO_2_ humidified atmosphere for 5 days. Splitting of neurospheres was performed by mechanical dissociation with a P1000 micropipette using 0.05% of trypsin in DMEM F12, followed by incubation for 3 min at 37°C; after trypsin inactivation with 10% FBS, the cells were centrifuged at 1,000 rpm and the supernatant discarded. Single cells were counted using a Neubauer chamber and plated in the same media with growth factors in 48-wells plates at a 1 cell/μL density, six wells per each condition and neurospheres were counted every 24 h for 4 days. Two independent experiments were performed.

### Confocal Imaging and Quantitative Analysis

To determine the number of proliferating neuroblasts (DCX^+^/BrdU^+^) and the number of newly-born granular neurons (Calb^+^/BrdU^+^) at the SGZ, sections from the dorsal hippocampus were used (Franklin and Paxinos, 2013). Six animals per genotype (Nenf^+/+^ and Nenf^−/−^) were assessed and four sections from each animal were analyzed. BrdU positive cells were identified using a confocal microscope (FV1000; Olympus, Hamburg, Germany) and the total number of double DCX^+^/BrdU^+^ and Calb^+^/BrdU^+^ cells was counted and normalized for the total number of BrdU positive cells and presented as a percentage.

### Contextual Fear Conditioning (CFC) Paradigms

For the behavior assessment, groups had the following composition: eight male Nenf^+/+^ and 10 male Nenf^−/−^. We performed a non-cued paradigm of the CFC where animals placed in an operant chamber (Med Associates) explored it for 3 min, after which three mild foot shocks (0.5 mA) were given randomly for the following 15 min. This conditioning phase was performed for two consecutive days. On the 3rd day, animals were placed for 3 min in the conditioned context (context A) followed by 3 min in a different context (context B; Fanselow and LeDoux, 1999; Anagnostaras et al., 2001; Mateus-Pinheiro et al., 2016; Ferreira et al., 2017). In this new context, a vanilla scent was added to the chambers and the chamber fan turned on; the walls were covered with patterned posters and the grid floor covered with white plastic. Each animal was scored for the percentage of time freezing in each chamber of the discrimination session day. Freezing behavior was scored manually and consisted of the total absence of movement besides regular breathing for more than 2 s. Parameters analyzed included the percentage of time freezing during the discrimination session, the total percentage of time freezing in contexts (A,B), and the index of discrimination between contexts as the ratio of percentage of time freezing (contexts A − B)/percentage of time freezing (contexts A+B).

### RNA Extraction, cDNA Synthesis and qPCR Analysis

For the gene expression study, the following groups of naïve mice were used: 10 male Nenf^+/+^ and 10 male Nenf^−/−^. Total RNA was isolated from DG samples using RNeasy Plus Micro Kit (Qiagen, Hamburg, Germany), following the manufacturer’s instructions. RNA quantification and quality were confirmed using the NanoDrop ND-1000 (NanoDrop, Thermo Scientific, Wilmington, DE, USA), and 500 ng of RNA from each sample was reverse transcribed into cDNA using the iScript cDNA Synthesis Kit (Bio-Rad Laboratories, Hercules, CA, USA) following the manufacturer’s instructions. Quantitative real-time PCR analysis (qPCR) was used to measure the expression levels of hypoxanthine guanine phosphoribosyl transferase (*Hprt*), 5-alpha-reductase 2 and 3 (*Srd5a2* and *Srd5a3*) and gamma-aminobutyric acid A receptor, delta (*Gabrd*).

Primers were designed using PrimerBlast software tool from National Center for Biotechnology Information (Bethesda, MD, USA), based in the respective GenBank sequences accession numbers; primers sequence are provided upon request. The qPCR reactions were conducted using equal amounts of cDNA from each sample and were performed in a Bio-Rad CFX96TM using SsoFast™ EvaGreen^®^ Supermix (Bio-Rad) according to the manufacturer’s instructions. The Hprt gene was used as an internal reference gene; its expression levels did not vary between experimental groups.

### Statistical Analysis

All quantitative data are expressed as Mean ± standard error mean (SEM). Data followed a Gaussian distribution, revealed by the normality test (using the Shapiro-Wilk test). Therefore, statistically significant differences between groups were determined using unpaired two-tailed Student’s *t*-test for two comparisons, and *Cohen’s d* for effect size is also presented. For the neurospheres clonal analysis, a two-way repeated measures ANOVA with a Bonferroni’s multiple comparisons post-test was used. *P-values* lower than 0.05 were considered statistically significant. All statistical procedures were performed using GraphPad Prism, version 7 (GraphPad Software Inc., San Diego, CA, USA).

## Results

### The Absence of NENF Impairs Cell Proliferation Only in the SGZ of the Hippocampus

**Figure 1:**
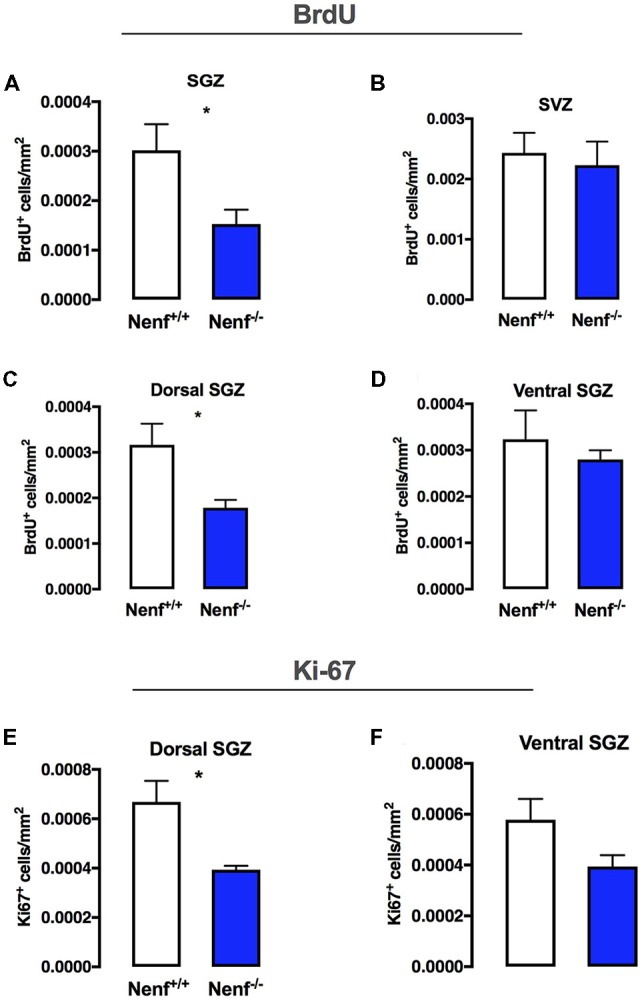
Neudesin ablation decreases proliferation selectively in the dorsal hippocampus. BrdU labeling for 24 h in the total sub-granular zone SGZ revealed that neuron-derived neurotrophic factor (NENF) ablation decreases proliferation **(A)**. NENF^−/−^ mice, when compared to the respective littermate controls, did not show proliferative rate differences in the subventricular zone (SVZ; **B**). When considering the SGZ divisions, dorsal **(C)** and ventral **(D)**, the effect of NENF is restricted to the dorsal pole although a slight decrease is also observed in the ventral pole of Nenf^−/−^ males but not significant. Ki67 positive cell counts in the dorsal **(E)** and ventral **(D)** SGZ, showed a selective significant decrease in proliferation of NENF^−/−^ mice in the dorsal area. Data presented as Mean ± SEM analyzed by two-tailed Student’s *t*-test. *p < 0.05.

To evaluate the effect of NENF ablation in the adult neurogenic niches, we first injected the DNA thymidine-analog BrdU 24 h before sacrifice, to assess dividing cells. We found that the absence of NENF (Nenf^−/−^ mice) reduces cell proliferation at the SGZ in ~50% when compared to controls (Nenf^−/−^ mice) [(*t*_(11)_ = 2.356; *d* = 0.29; *p* < 0.05); Figure 1A]. In the SVZ proliferation did not differ between genotypes (Figure 1B).

The hippocampal formation is known to have topographical specificities (Pinto et al., 2015); thus we evaluated SGZ proliferation in dorsal and ventral poles. We observed lower levels (45% decrease when compared to wild-type mice) of proliferation in the dorsal SGZ of Nenf^−/−^ mice [(*t*_(10)_ = 2.405; *d* = 1.52; *p* < 0.05); Figure 1C]; we did not observe significant differences in proliferation between genotypes in the ventral SGZ, despite a trend for less BrdU^+^ cells in the absence of NENF (Figure 1D). Using Ki67, a standard endogenous marker for proliferation index, we observed a significant decrease in Ki67^+^ cells in the dorsal SGZ of Nenf^−/−^ mice when compared to littermate controls [(*t*_(6)_ = 3.14; *d* = 2.22; *p* < 0.05); Figure 1E]. No significant differences were observed in the ventral SGZ, although, similarly to BrDU, a trend for a decrease in Ki67^+^ cells is observed (Figure 1F).

### NENF Absence Impairs *in vitro* Hippocampal Neural Stem Cells Self-Expansion

**Figure 2:**
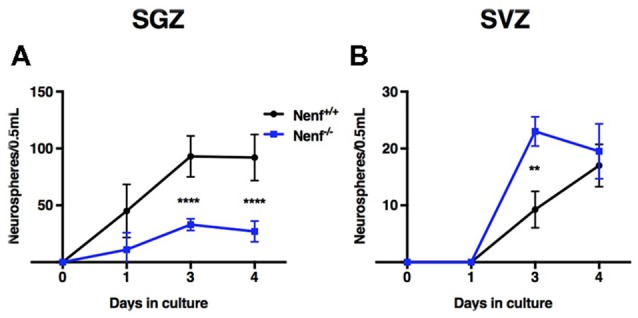
*In vitro* hippocampal derived neural stem cells (NSCs) impaired self-renewal capacity in the absence of NENF. Primary neurospheres obtained from male pups were counted at days 1, 3 and 4 of culture. NSCs derived from hippocampus (SGZ) of NENF^−/−^ mice formed significantly less spheres when compared with NENF^+/+^ **(A)** but not in NSCs derived from SVZ (**B**). Data presented as Mean ± SEM; analyzed by two-way repeated measures ANOVA with a Bonferroni’s multiple comparisons post-test. ****p < 0.0001 for Nenf^+/+^ vs. Nenf^−/−^ for days 3 and 4 in the SGZ and **p < 0.001 for Nenf^+/+^ vs. Nenf^−/−^ for days 3 in the SVZ.

*In vitro* neurosphere cultures were performed to confirm the *in vivo* proliferation deficits observed in Nenf^−/−^ mice. Postnatal derived stem cells were isolated from the hippocampal DG and the SVZ of Nenf^+/+^ and Nenf^−/−^ mice, and their self-renewal capacity evaluated in a clonal neurosphere assay. We observed a significant 3-fold decrease in the total number of primary neurospheres formed from Nenf^−/−^ when compared to Nenf^+/+^ cells in days 3 and 4. Analysis of the neurosphere counts along the days showed a significant interaction between variables [(F_(3,21)_ = 14.68 η^2^ = 0.15 *p* < 0.0001); Figure 2A], with a major effect of genotype (F_(1,7)_ = 48.99 η^2^ = 0.26 *p* < 0.01), and of time (F_(3,21)_ = 48.25 *η*^2^ = 52.16 *p* < 0.01); Bonferroni *post hoc* tests showed significant differences between genotypes at days 3 and 4 (*p* < 0.0001). Day 3 revealed the most significant difference between genotypes SGZ [(*t*_(7)_ = 9.19; *d* = 0.92; *p* < 0.00001; Figure 2A. Differences at day 3 in the SVZ were also present, although Nenf^−/−^ mice showed significantly more neurospheres then controls [(*t*_(5)_ = 4.89; *d* = 3.66; *p* < 0.001; Figure 2B]. On the other hand, we did not observe differences between genotypes in SVZ-derived neurospheres counts along the days (Figure 2B). This finding confirms the specificity (SGZ but not SVZ) of NENF’s neurotrophic modulation observed *in vivo* in the adult brain.

### The Absence of NENF Impairs the Differentiation of SGZ Adult Progenitors

**Figure 3:**
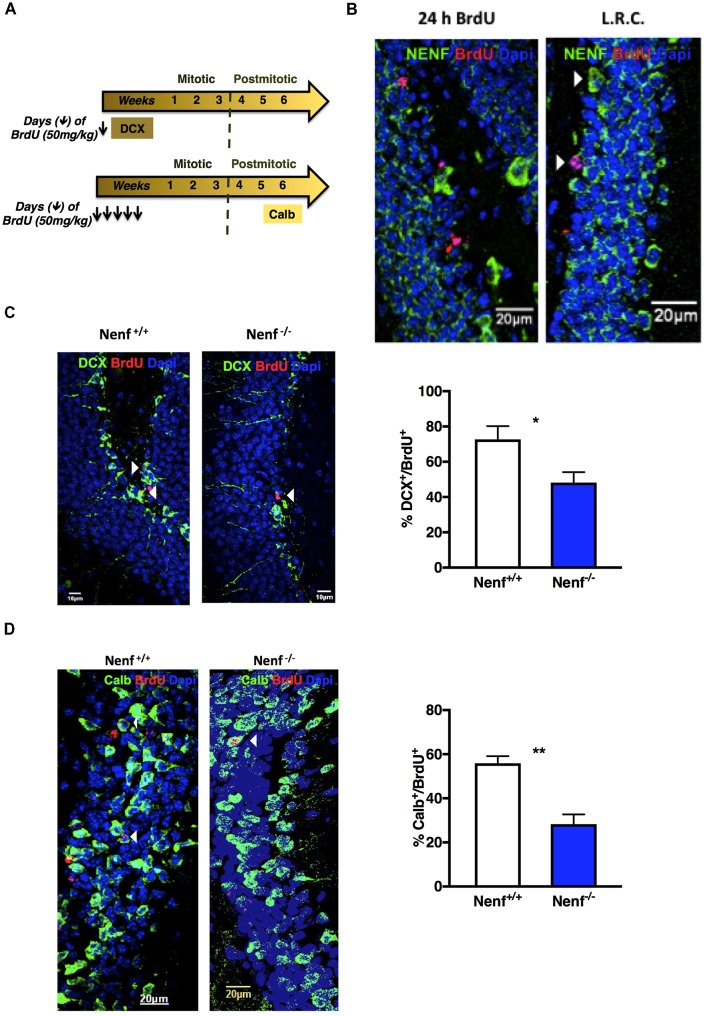
Ablation of NENF impairs neural committed cell proliferation, survival and maturation in the adult hippocampus. Schematic representation of hippocampal neurogenesis chronology and 5-bromo-2′-Deoxyuridine (BrdU) protocol, including specific markers for cellular types **(A)**. NENF is not expressed in 24 h labeled BrdU neuroblasts, its expression appears later in newly born granular neurons, as seen with NENF (green) and BrdU label retaining cells (LRC) for 7 weeks (red; **B**). Co-labeling of doublecortin (DCX) and BrdU in Nenf^+/+^ and Nenf^−/−^ animals is represented and proliferative neuroblasts counting in the SGZ of NENF^+/+^ and NENF^−/−^ showed a significant decrease in the absence of NENF **(C)**. BrdU followed by 7 weeks of chase period identifies adult newborn granular neurons in the dentate gyrus (DG) by co-labeling with a neuron mature marker calbindin (Calb), as represented for Nenf^+/+^ and Nenf^−/−^ animals. There was a significant reduction in the percentage of BrdU positive cells that co-label Calb in NENF^−/−^ **(D)**. Data presented as Mean ± SEM analyzed by two-tailed Student’s *t*-test *p < 0.05 and **p < 0.01. White arrow indicated BrdU^+^ cells co-localizing with the respective markers.

The diminished proliferation observed in the dorsal SGZ of Nenf^−/−^ mice prompted us to further characterize neurogenesis specifically in the dorsal pole of the hippocampus. Proliferative neuroblasts were labeled with both DCX and BrdU (24 h; BrdU^+^/DCX^+^; Figure 3A). The proliferative deficits observed in the absence of NENF seem to result from an alteration in the neuroblasts population, since Nenf^−/−^ mice SGZ displayed 24% less DCX^+^/BrdU^+^ cells when compared to control littermates [(*t*_(8)_ = 2.57; *d* = 2.71; *p* < 0.05); Figure 3C]. Of notice, NENF protein expression was not detected in 24 h BrdU^+^ cells but its expression in BrdU^+^ is detected later in differentiation, after 7 weeks of BrdU labeling (Figure 3B). Thus, to assess differentiation we chose a late neuronal maturation marker (Calb) that begins its expression after 4 weeks of differentiation, instead of a global neuronal marker such as NeuN, to evaluate BrdU^+^ cells and assess late stages of differentiation. Herein, we estimated newborn neurons in the DG by performing BrdU labeling for five consecutive days and analyzed the DG after 7 weeks (Figure 3A). In the absence of NENF, there is a significant 1.8-fold decrease in the number of newborn neurons (Calb^+^/BrdU^+^) in the DG when compared to control mice [(*t*_(6)_ = 5.04; *d* = 0.35; *p* < 0.01); Figure 3D].

### Lack of NENF Influences Hippocampal Function in a Fear Conditioning Behavior Task

**Figure 4:**
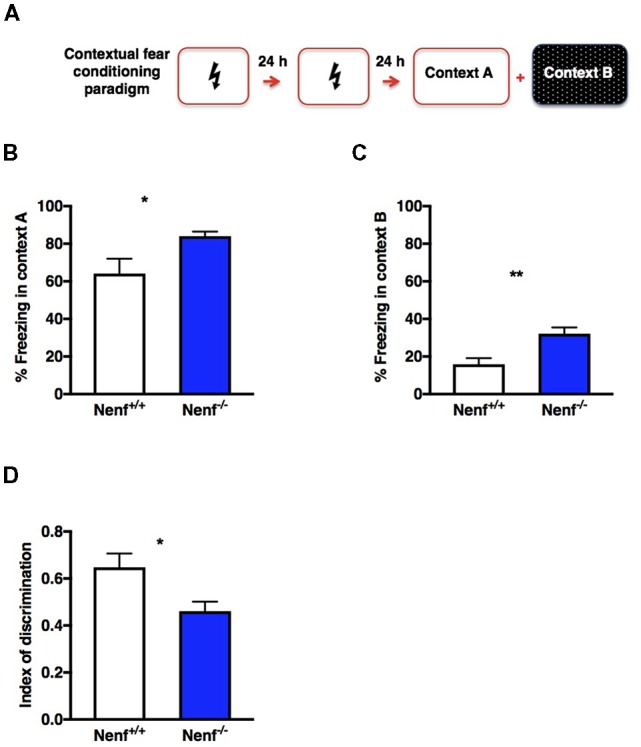
Hippocampal dependent memory impaired in the absence of NENF. An experience-dependent contextual fear conditioning (CFC) discrimination paradigm was performed **(A)**. NENF^−/−^ mice showed more freezing in the aversive context (context A) compared to controls **(B)** this difference is also present in the novel context (context B; **C)**. A decrease index of discrimination between aversive and novel contexts is observed in NENF^−/−^ when compared to Nenf^+/+^ **(D)**. Data presented as Mean ± SEM analyzed by two-tailed Student’s *t*-test *p < 0.05 and **p < 0.01.

Adult hippocampal neurogenesis was implicated in dorsal hippocampal related behaviors, such as pattern separation (Saxe et al., 2006). Animal models with negative modulation of adult hippocampal neurogenesis display impairments in CFC paradigms involving the discriminations of similar contexts (Saxe et al., 2006; Sahay et al., 2011). We used a non-cued CFC paradigm to test dorsal hippocampal function based on lesion studies that show significantly less involvement of the amygdala compared to cued CFC paradigms (Anagnostaras et al., 2001). A two-session conditioning phase was chosen to ensure that both genotypes discriminate the shock-context association (Figure 4A). The animals were positively conditioned since all froze significantly more in the aversive context (context A) than in the new context (context B). Nenf^−/−^ mice froze significantly more in both contexts [context A: (*t*_(10)_ = 2.235; *d* = 0.453; *p* < 0.05), Figure 4B; and context B: (*t*_(11)_ = 3.484; *d* = 0.28; *p* < 0.01), Figure 4C] indicating a generalized exacerbation of the fear response in the absence of NENF. Ultimately, discrimination between contexts revealed that in the absence of NENF, animals show a significant inferior index of discrimination, [(*t*_(10)_ = 2.62; *d* = 0.84; *p* < 0.05); *p* < 0.01); Figure 4D]. These results suggest that there is a moderate decrease in contextual discrimination revealing a cognitive impairment in the absence of NENF.

### Progesterone Metabolism-Related Gene Expression in the DG of NENF^−/−^ Mice

**Figure 5:**
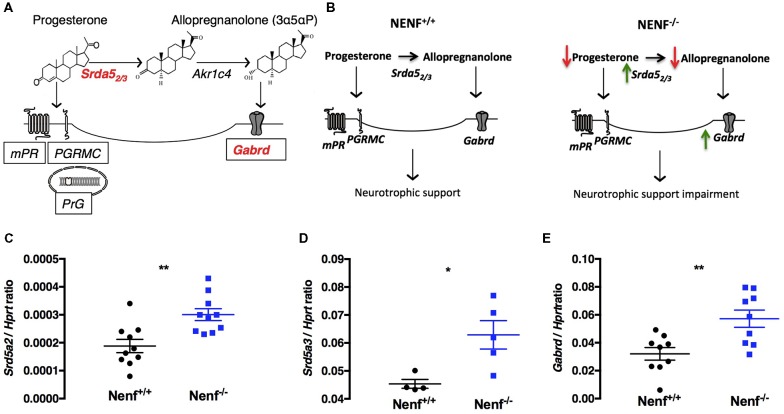
Ablation of NENF alters steroid metabolism in the DG. Schematic representation of progesterone metabolization into neurosteroids and the respective binding receptors **(A)**. Proposed mechanism for the role of NENF neurotrophic support in the DG **(B)**. Since NENF is a membrane-associated progesterone receptor family (MAPR) protein, progesterone metabolism gene expression was assessed in the DG. *Srd5a2* and *Srd5a3* were found increased in Nenf^−/−^ **(C,D)**. Progesterone is the main source of active steroids in the brain that bind to GABA A receptors with a delta subunit, we aobserved an overexpression of *Gabrd* gene in the DG in the absence of NENF **(E)**. Data presented as Mean ± SEM; analyzed by two-tailed Student’s *t*-test *p < 0.05 and **p < 0.001. Hypoxanthine guanine phosphoribosyl transferase (*Hprt*), 5-alpha-reductase 2 and 3 gene (*Srd5a2* and *Srd5a3*) and gamma-aminobutyric acid A receptor, delta gene (*Gabrd*), 3α-hydroxysteroid dehydrogenase gene (*AKR1C4*), progesterone membrane receptor (mPR), progesterone receptor membrane component 1(PGRMC1), nuclear progesterone receptor (PrG).

Progesterone metabolization through 5α-reductase results in neurosteroids synthesis, which by allosteric binding to delta^+^ GABA A receptors sustains the excitability/inhibition balance in the DG, modulating tonic inhibition (Figure 5A). On the other hand, NENF was suggested to play a role in the progesterone stabilization complex to potentiate the activation of membrane progesterone receptors (Kimura et al., 2013). Given the potential interaction between NENF and progesterone-related signaling, we evaluated if genes related to progesterone metabolism could be impaired in the DG of Nenf^−/−^ mice (Figure 5A). We observed that the expression of the isoform 2 of 5α-reductase (*Srd5a2*) gene is significantly increased (50% higher) in the absence of NENF [(*t*_(18)_ = 3.53; *d* = 0.73; *p* < 0.01; Figure 5C]. We observed a similar increase in the expression of the isoform 3 of 5α-reductase (*Srd5a3)* [(*t*_(7)_ = 2.95; *d* = 1.58; *p* < 0.05); Figure 5D], revealing a sharp difference between genotypes in the expression of the progesterone catabolism enzyme. Furthermore, we tested the gene expression of delta subunit of the GABA A receptor because it is modulated by the action of progesterone metabolites (via 5α-reductase) in the DG. We found a significant 1.8-fold increase in the expression of the *Gabrd* gene [(*t*_(16)_ = 3.28; *d* = 1.55; *p* < 0.01); Figure 5E], indicative of a potential alteration in the DG tonic inhibition control. Together these results suggest that the proposed mechanism for NENF neurotrophic support in the DG can be through progesterone availability for metabolization into neurosteroids (Figure 5B).

## Discussion

Herein, we provide the first data concerning the characterization of the impact of the ablation of NENF in the adult neurogenic niches (SVZ and SGZ). Noticeably, the absence of NENF in adult mice impairs the proliferation and differentiation of neuronal progenitor cells; this effect is specific to the dorsal SGZ of the hippocampus. In accordance, we confirmed *in vitro* that neural stem cells isolated from the hippocampus of postnatal neudesin-null mice form fewer neurospheres than the ones from control mice. Of interest, the changes in the DG correlate with alterations in a hippocampal-dependent behavior, as evaluated in the CFC paradigm. Furthermore, the observations indicated above might be related to altered neurosteroids metabolism/signaling.

The potent neurotrophic properties of NENF were initially demonstrated in cultures of embryonic neural precursor cells and neurons (Kimura et al., 2005). We now confirm that the neurotrophic properties of NENF are also relevant for the adult neurogenic niches. Interestingly, NENF influences the SGZ neurogenic niche solely, since its absence yields a significant reduction in cell proliferation and ultimately leads to decreased neurogenesis in the dorsal hippocampus, but not in the SVZ. This result might not be surprising since although the cellular populations of these niches share similarities, they are regulated in distinct ways, namely the response to extrinsic signals (Seaberg and van der Kooy, 2002). Thus, NENF, *per se* as a neurotrophic factor, or indirectly by modulating other signaling pathways (as will be discussed later) exerts an effect solely on the SGZ neurogenic niche. Importantly, the methodological approach used here only permits determining the impact of NENF in the number of fast-dividing cells and of newborn neurons. Thus, other relevant intermediate stages of SGZ neurogenesis, not addressed in this work should be studied to explore in further detail the impact of NENF in the hippocampal NSCs niche dynamics.

Of interest, we found that the impairment in neurogenesis observed in the absence of NENF is restricted to the dorsal hippocampus. It is not the first time that we find a hippocampal asymmetry in mice with ablation of the Nenf gene. Previously we reported that DG granular cells of the ventral but not the dorsal hippocampus are significantly atrophied when NENF is ablated; this phenotype was strongly correlated with the anxiety-like behavior presented by neudesin-null mice (Novais et al., 2013). Of notice, newborn granular neurons from the dorsal DG (since early stages of maturity) play a significant role in hippocampal-dependent behaviors, namely the CFC paradigm (Saxe et al., 2006; Kheirbek and Hen, 2011, 2014; Sahay et al., 2011). When submitted to the CFC behavioral task, mice with ablation of NENF showed alterations in contextual discrimination. While the alterations in neurogenesis and the CFC described here are concomitant, we cannot discriminate if this behavioral phenotype is explicitly related to dorsal DG proliferation deficits, or if it also stems from the integration of non-functional mature neurons in the hippocampal circuitry.

The mechanism by which NENF impacts the number of newborn granular neurons is not yet understood and deserves further investigation. Newly formed neurons migrate to the granular zone of the DG and project an axon to the CA3 layer of the hippocampus, thus integrating into the hippocampal circuitry (Zhao et al., 2008; Piatti et al., 2011). This process is highly modulated by neurotrophins and in particular by BDNF (Lessmann, 1998; Lu, 2003). Interestingly an interaction between NENF and BDNF was previously described namely the anorexigenic effects promoted by recombinant NENF in the hypothalamus occur through BDNF (Byerly et al., 2013), and NENF was proposed to be involved in the regulation of appetite circuitry by BDNF (Byerly et al., 2013; Ohta et al., 2015). Thus, it is possible that NENF impact on hippocampal neurogenesis also occurs via the modulation of BDNF. Another possible mechanism that can lead to decreased neurogenesis in the absence of NENF might be related to the activation of the non-genomic effects of progesterone. This possibility is based on the fact that NENF shares ~40% structural homology with PGRMC1. Indeed, Kimura et al. previously discussed the interaction between NENF and progesterone and how this interplay could relate to the neurotrophic support ascribed to NENF; this involves the NENF mediated presentation of progesterone for membrane progesterone receptor activation (Kimura et al., 2013). In fact, the activation of non-genomic cascades through the membrane progesterone receptor PGRMC1, such as MAPK/ERK or PI3K pathways, regulates proliferation of neural progenitors, involving progesterone in the modulation of NSCs *in vitro* (Liu et al., 2009). In the adult hippocampus, the main effects mediated by progesterone occur through its metabolization (via 5α-reductase) into neurosteroids, namely allopregnanolone (Reddy, 2010). For instance, the blockage of 5α-reductase with finasteride reduces proliferation with no influence in cell survival (Römer et al., 2010), an effect attributed to a decreased synthesis of neurosteroids. Of interest, herein, in the absence of NENF, the expression of 5α-reductase increases. This response might represent a compensatory mechanism to counteract the decrease in the local availability of its substrate due to the absence of NENF.

Binding of neurosteroids to δGABA_A_ receptors modulates neuronal tonic inhibition and alterations in this receptors activation might contribute to an imbalanced synaptic excitation/inhibition in the DG (Lee et al., 2016). Furthermore, the constitutive ablation of δGABA_A_R leads to impairment in the CFC paradigm and decreases neurogenesis (Whissell et al., 2013). Herein we observe increased expression of δGABA_A_R, concomitant with decreased neurogenesis in the absence of NENF, which opposes the above described (Whissell et al., 2013). Nevertheless the effect of the absence of NENF in the expression of δGABA_A_R might relate to neurosteroids availability to activate δGABA_A_R mechanisms.

Overall, herein we show that NENF modulates cell proliferation and neurogenesis and concomitantly impacts hippocampus-dependent behavior. Nevertheless, these observations should be taken carefully given that the Nenf-null model used in this study has a constitutive gene deletion. Thus, further research on the impact of the ablation of Nenf in brain development should be addressed in the future.

## Author Contributions

AN, AS, ACF, AMF and FM performed the experiments. AN and JS analyzed the data. NS, JP and JS supervised the study. AN, FM and JS wrote the manuscript. All authors revised the manuscript.

## Conflict of Interest Statement

The authors declare that the research was conducted in the absence of any commercial or financial relationships that could be construed as a potential conflict of interest.
